# Highly Dispersed Few-Nanometer Chlorine-Doped SnO_2_ Catalyst Embedded in a Polyaniline Matrix for Stable HCOO^–^ Production in a Flow Cell

**DOI:** 10.1021/acsami.2c12428

**Published:** 2022-09-09

**Authors:** Daniele Sassone, Juqin Zeng, Marco Fontana, M. Amin Farkhondehfal, Candido F. Pirri, Sergio Bocchini

**Affiliations:** † Center for Sustainable Future Technologies (CSFT)@Polito, Istituto Italiano di Tecnologia, Via Livono 60, 10144 Torino, Italy; ‡ Department of Applied Science and Technology-DISAT, Politecnico di Torino, Corso Duca degli Abruzzi 24, 10129 Torino, Italy

**Keywords:** CO_2_RR, binder-free electrodes, formate production, Cl-doped SnO_2_, nanocatalysts, electrochemistry

## Abstract

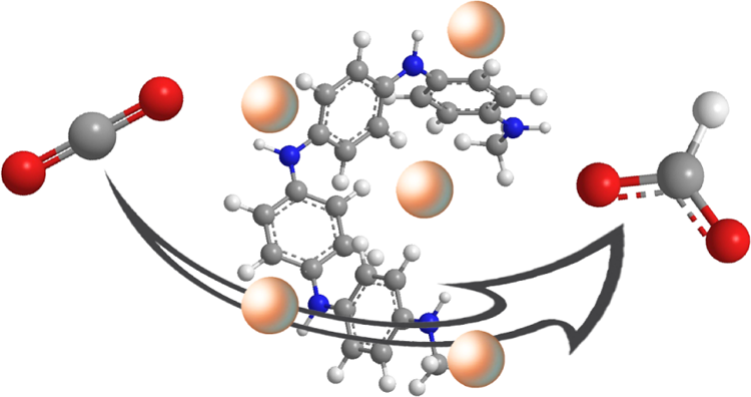

With the spread of alternative energy plants, electrolysis processes are becoming the protagonists of the future industrial generation. The technology readiness level for the electrochemical reduction of carbon dioxide is still low and is largely based on precious metal resources. In the present work, tin ions are anchored on a polyaniline matrix, via a sonochemical synthesis, forming a few atomic layers of chlorine-doped SnO_2_ with a total loading of tin atom load of only 7 wt %. This catalyst is able to produce formate (HCOO^–^) with great selectivity, exceeding 72% of Faradaic efficiency in the first hour of testing in 1 M KHCO_3_ electrolyte, with a current density of more than 50 mA cm^–2^ in a 2 M KHCO_3_ electrolyte flow cell setup. Catalyst stability tests show a stable production of HCOO^–^ during 6 h of measurement, accumulating an overall TON_HCOO^–^_ of more than 10,000 after 16 h of continuous formate production. This strategy is competitive in drastically reducing the amount of metal required for the overall catalysis.

## Introduction

The electrochemical reduction of carbon dioxide into fuels and chemicals represents an important technology which is able to store the excess energy from green alternative energy plants during low demand hours.^1,2^ The carbon dioxide reduction reaction (CO_2_RR) is an extremely interesting but demanding process. Operating current densities and cell voltages must be above 200 mA cm^–2^ and below 3 V, respectively.^3,4^ Moreover, the overall economic viability of the process could be sustained when the price of each component decreases. Among the available CO_2_RR products (CO, HCOO^–^, CH_4_, CH_3_OH, C_2_H_4_, CH_3_CH_2_OH, etc.),^3^ the electrochemical production of formate and carbon monoxide is the technology closest to industrialization.^3^ A green technology that is able to produce a large amount of formate is of great interest for its use in various fields, such as in the agriculture activities (as a preservative and antibacterial agent in livestock feed, present in various fermentation processes^5^), in various industrial productions (such as the preparation of textile, paper, and rubber), and in frontier technologies such as the formic acid fuel cell.^6^ Although the main obstacle is still the price of energy,^1^ the high cost of the catalyst and membrane preparation, including product purification, largely contributes to the final price. Cathodic catalyst optimization is still a very active research topic with little knowledge and great potential growth. The benchmark heterogeneous catalysts for the cathodic CO_2_RR are metal/metal oxide nanoparticles.^7,8^ Since the presence of a metal active site is essential to perform a potential-accessible reduction of CO_2_, in order to keep the price down, the number of metal atoms required to form the active sites should be minimized.^9^ The ideal design is the one of single-metal-atom materials^10,11^ in which, similar to the metal–organic compounds,^12−14^ the active site is a single metal atom or cation stabilized in a heterogeneous material or complexed with molecular ligands. The most common strategy is the design of defected catalysts that achieve the smallest possible cluster size,^15−17^ thus maximizing the number of active sites per atoms involved in the cluster. Regardless of the chosen strategy, there is little knowledge about catalyst changes under potential during catalysis, especially for electrocatalysis. Cations can be reduced and become moveable on the catalyst surface, creating clusters of few atoms and, as a result, sintered into larger nanoparticles.^18^

In a previous work, we studied several doped polyanilines with different cations (Sn, Mn, Cu, and Fe) in order to form single-metal-atom materials for the electrochemical reduction of CO_2_.^10^ In this work, we started with a similar approach of Sn cations doping on a polyaniline material. During both the polymerization and doping steps, the reaction was performed under intense sonochemical treatment inducing locally high-temperature peaks. This harsh condition led to the synthesis of nanosize polyaniline matrix in which extremely small particles of SnO_2_, on the order of few atomic layers, were incorporated without the addition of any expensive binder. As a consequence of the high defects present on such small SnO_2_ nanoparticles, a chloride anion doping occurs, in which all along the oxide, the chlorine anions are incorporated. The composite material was evaluated in a flow cell setup for the reduction reaction of carbon dioxide (CO_2_RR) showing interesting performance as a catalyst for the formate production. The great selectivity and important stability, over several hours of test, highlighted the innovative approach to minimize the amount of metal required to catalysis, drastically reducing the overall price. The catalyst as prepared was extremely active considering the tremendous low amount of metal employed. The synthetic procedure is easily scalable, also considering the low price required to produce the polymer support comparing it to the extremely expensive reference fluorinated binders.

## Results and Discussion

### Reagents

All the reagents were purchased from commercial sources without further purification. *N*-Phenyl-1,4-phenylenediamine (DANI, 98%), tin(IV) chloride (SnCl_4_·5H_2_O, 98%), ammonium persulfate [APS, (NH_4_)_2_S_2_O_8_, 98%], methanol (MeOH, 99.9%), *N*-methyl-2-pyrrolidinone (NMP, 99.5%), and fuming hydrochloric acid (HCl, 37%) were all purchased from Merck.

### Synthesis

In this work, 1.0 g of DANI (5.43 mmol, 184.24 g mol^–1^) was dissolved in 15 mL of 70:30 v/v MeOH/H_2_O with 1% w/w of HCl solution and kept under sonication for 10 min in a 10 °C bath. Then, 1.86 g of APS (8.15 mmol, 228.18 g mol^–1^) dissolved in 15 mL of the same solution was added in the former dropwise along 5 min with continuous sonication. Once the two solutions were completely mixed, they were kept under sonication for further 3 h in a cold bath and then at room temperature (RT). The solution was then centrifuged and washed twice with Milli-Q water and a last time with MeOH. The final green powder was then immersed in 10 mL of 32% ammonia solution and stirred for one night. The obtained blue powder was then centrifuged and washed three times with water before placing in an oven overnight to dry. The polymer obtained is named EB-PANI. Successively, 370 mg of EB-PANI (4 mmol, PM 93.13 g mol^–1^) was dissolved with 2.1 g of SnCl_4_·5H_2_O (6 mmol, PM 350.60 g mol^–1^) in 20 mL of MeOH in a sealed vial. The solution was stirred at 60 °C for the night. The solution was precipitated by adding 20 mL of water and then centrifuged. The powder was washed and centrifuged three times with water to remove any trace of metal salts. The obtained powder is labeled as SnO_2_/PANI.

### Physical and Chemical Characterizations

**Figure 1:**
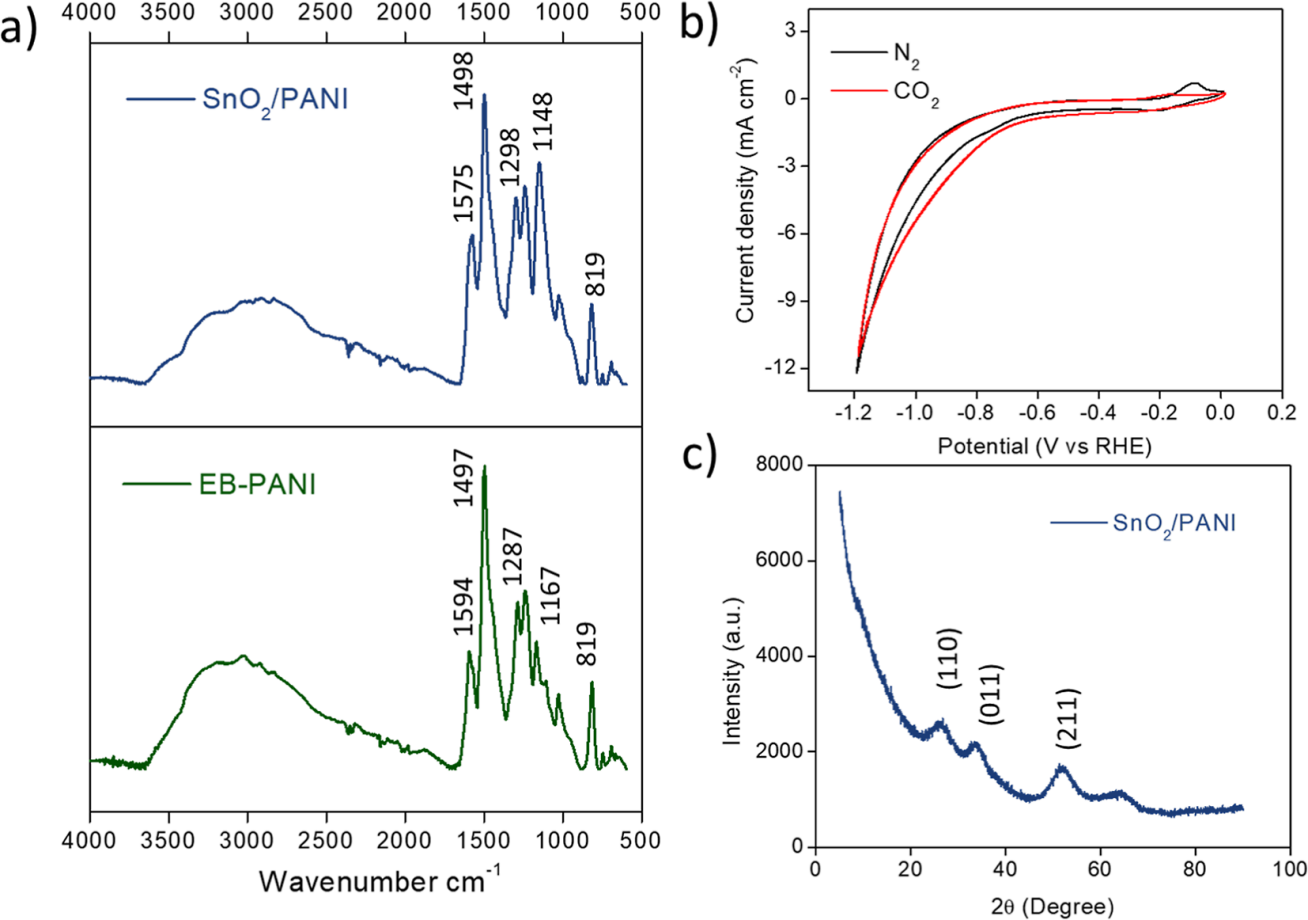
(a) ATR spectra of SnO_2_/PANI (blue) and EB-PANI (green). (b) Cyclic voltammetry of the SnO_2_/PANI sample in 0.1 M KHCO_3_ electrolyte under a N_2_ atmosphere (black line) and CO_2_ atmosphere (red line). (c) XRD pattern of the SnO_2_/PANI sample.

The correct polymerization of the polyaniline is evaluated using the attenuated total reflectance (ATR) technique. The spectra of EB-PANI and SnO_2_/PANI are reported in Figure 1a. In both samples, a similar stretching pattern is recorded, which shows the correct polymerization of the polyanilines. The C=N stretching of quinoid diimine unit appears at 1594 and 1575 cm^–1^, respectively, for EB- and SnO_2_/PANI, evidencing a shift for the latter in presence of tin ions interacting with this chemical group. The C–C aromatic ring stretching of the benzenoid diimine unit at 1497 cm^–1^ is present in both samples, and the C–N stretching of aromatic amine is registered at 1287 and 1298 cm^–1^ for EB- and SnO_2_/PANI, respectively, with a further shift induced by tin ion interaction. At 1167 cm^–1^, there is the stretching of the quinoid ring, and at 819 cm^–1^, there is the out-of-plane stretching due to hydrolysis of immine units of the polymer.^19^ All the signals are coherent with the published literature.^20^

Figure 1b shows the electrochemical behavior of the SnO_2_/PANI catalyst studied by cyclic voltammetry (CV). The CV tests were performed in a 0.1 M KHCO_3_ solution to ensure a stable pH buffer and adequate electrical conductivity. Before each measurement, the electrolyte solution was purged for 30 min with a proper gas (first N_2_ and then CO_2_) and the bulk pH was measured. The applied potential is relative to the Ag/AgCl reference electrode during the measurements, and then the reported potential was converted to the reversible hydrogen electrode (RHE) potential in order to be compared. As can be seen in Figure 1b, the black line referring to the N_2_ atmosphere shows a great reversible reduction peak around −0.2 V versus RHE, typical of the SnO_2_ species. Switching to CO_2_ atmosphere instead, this peak shifts toward more negative potential, in parallel with a higher current density across the potential than that of N_2_, suggesting a better selectivity toward the CO_2_RR rather than the competitive HER. The presence of SnO_2_ deposition on the PANI polymer is perfectly evidenced in Figure 1c, in which the powder X-ray diffraction (XRD) pattern of the SnO_2_/PANI sample powder shows the well-known diffraction of tin oxide species (see for example crystallography open database ID: 2101853, http://www.crystallography.net/cod/index.php).

**Figure 2:**
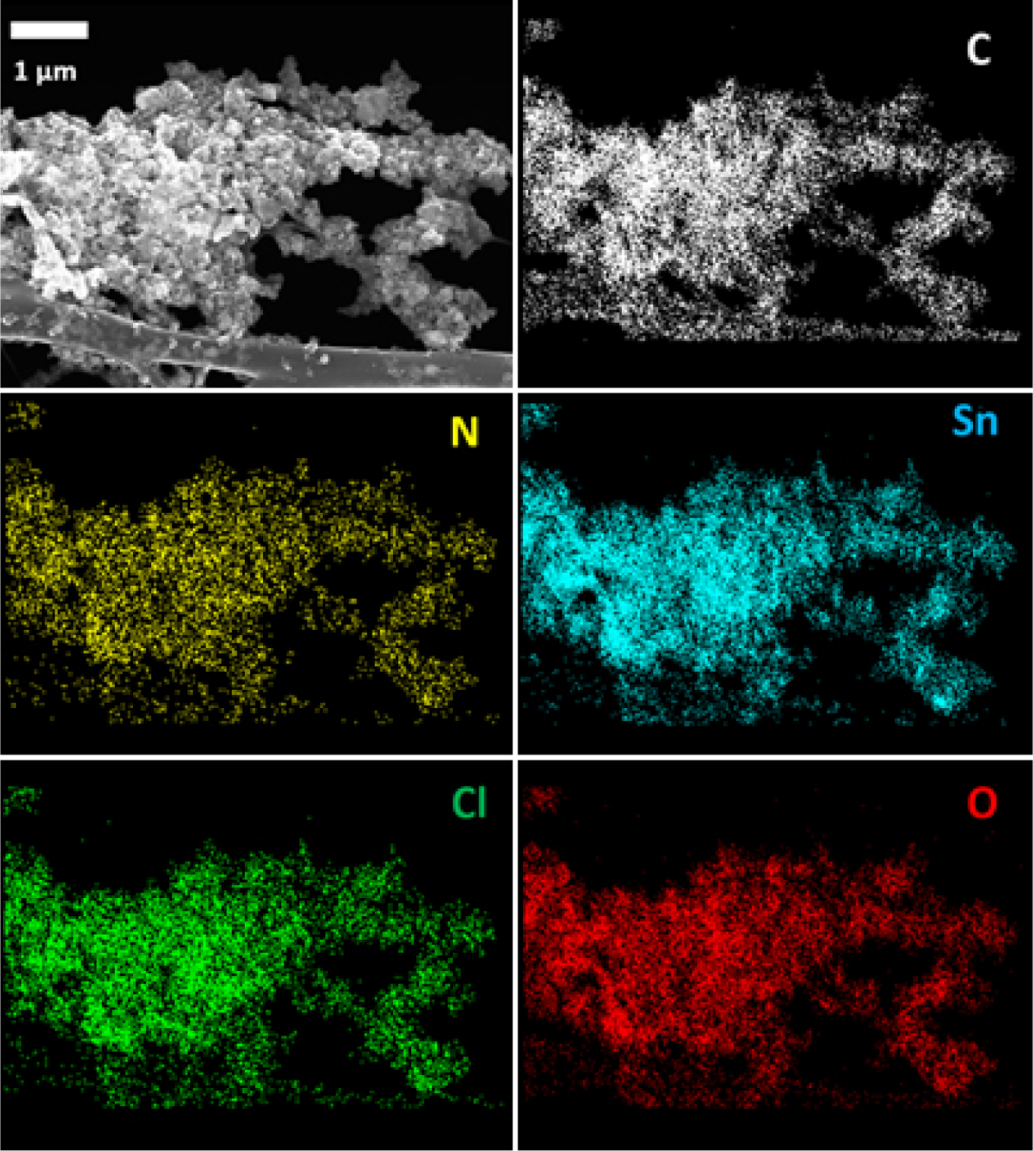
Electron diffraction image (top left) and EDX maps of C, N, Sn, Cl, and O chemical elements for the SnO_2_/PANI sample.

Further insights into the successful insertion of the tin-containing nanostructures into the PANI matrix are provided by energy-dispersive X-ray (EDX) analysis spectroscopy inside field-emission scanning electron microscopy (FESEM), which qualitatively confirms the expected chemical composition for the catalyst and demonstrates the homogeneous decoration at the submicrometric scale of the polymer with the Sn-containing structures (Figure 2). Interestingly, EDX mapping shows also a homogeneous displacement of chlorine atoms all over the nanoparticles. Due to the extreme low size of the tin oxide nanoparticle, the chloride anions compensate for the surface charge formed by the crystal subcoordinated tin atoms, causing partial doping. This phenomenon is similar to how tin oxide perovskite solar cell surface is treated to cause chloride doping in order to tune the cell’s performance.^21−23^

**Figure 3:**
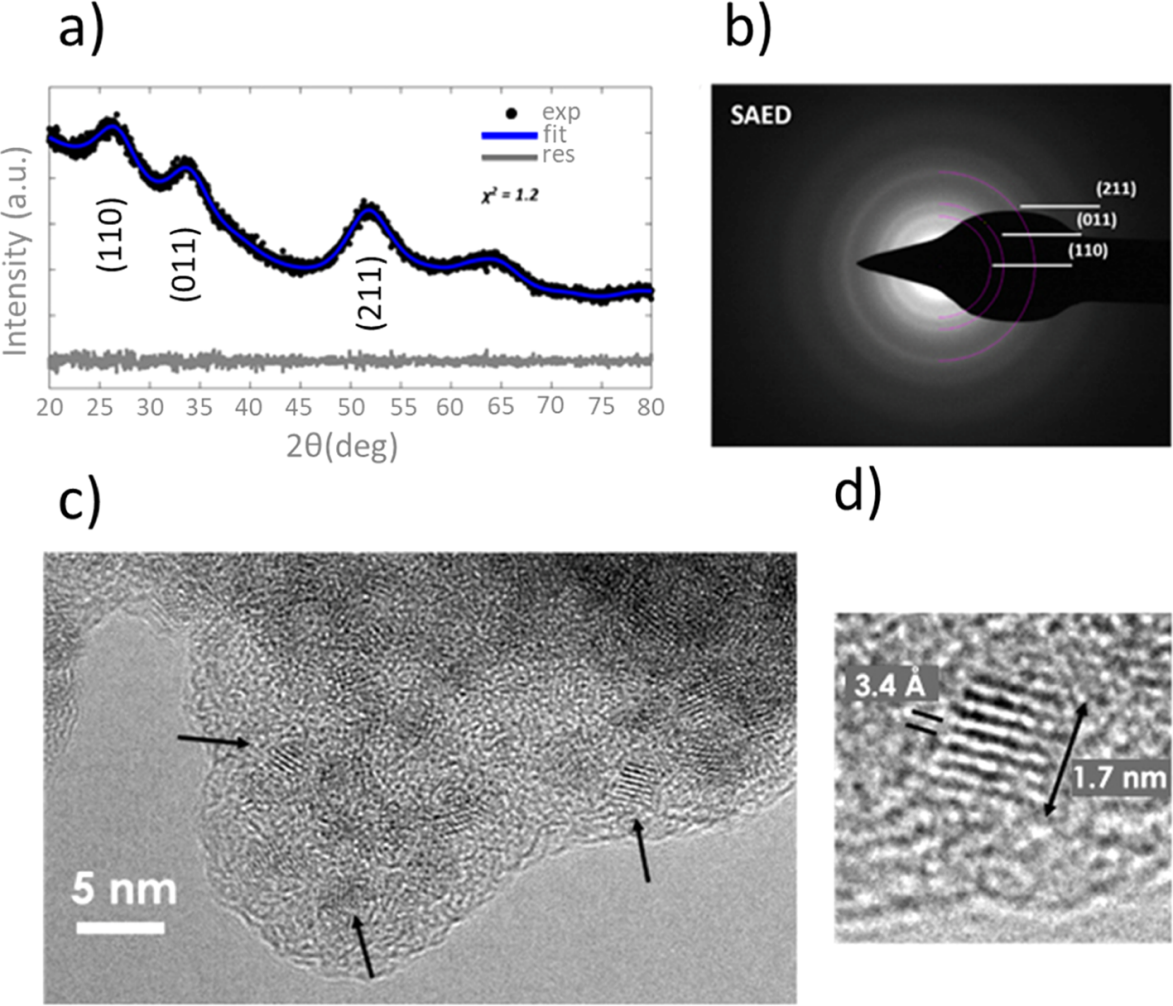
SnO_2_/PANI morphological characterization. (a) XRD experimental data, alongside fit through Paley refinement and the corresponding residuals. (b) SAED and (c,d) HR-TEM images. The arrows in (c) point to the crystalline domains. The interplanar spacing measured in (d) corresponds to the (110) family of planes in rutile SnO_2_.

A careful characterization of the morphology and structure of the SnO_2_/PANI catalyst was obtained by combining XRD with electron microscopy techniques, as shown in Figure 3. To obtain quantitative information from the XRD data, Pawley refinement was employed, as shown in Figure 3a. The XRD pattern clearly shows very broad peaks, which correspond to the tetragonal unit cell (*a* = *b* = 4.761 Å, *c* = 3.198 Å) with space group *P*42/*mnm* (# 136), in accordance with the crystalline structure of rutile SnO_2_ (see for example crystallography open database ID: 2101853, http://www.crystallography.net/cod/index.php). Since there are no contributions from the EB-PANI,^9^ XRD data suggest that the SnO_2_/PANI catalyst consists of an amorphous polymeric matrix, decorated with nanometric SnO_2_-like structures (∼1.5 nm in size, based on Pawley refinement).

Both morphology and structure were finally investigated with transmission electron microscopy (TEM). Structural information from selected area electron diffraction (SAED, in Figure 3b) confirms XRD results, showing contributions compatible with the rutile SnO_2_ crystalline structure. High-resolution TEM (HR-TEM) images (Figure 3c) provide useful details at the nanometric scale. Specifically, it is possible to directly visualize the amorphous polymer matrix homogeneously decorated with very small crystalline domains (size typically <2 nm). Measuring the interplanar spacing from HR-TEM images (Figure 3d), the rutile SnO_2_ structure is confirmed. The visualization of these extremely small nanoclusters homogeneously dispersed all around the polyaniline highlights the novelty of the tin oxide incorporation on a polymer matrix, which prevents the metal oxide from agglomerating into larger structures. In such low-size nanoclusters, the ratio of the number of atoms actually exposed to the total number required to construct the active site is of great interest for a more scalable commercial process. In conclusion, such characteristics evidence the extremely low use of tin atoms to construct the catalytic site and thus the benefits of a polymer matrix as support for the catalyst preparation.

**Figure 4:**
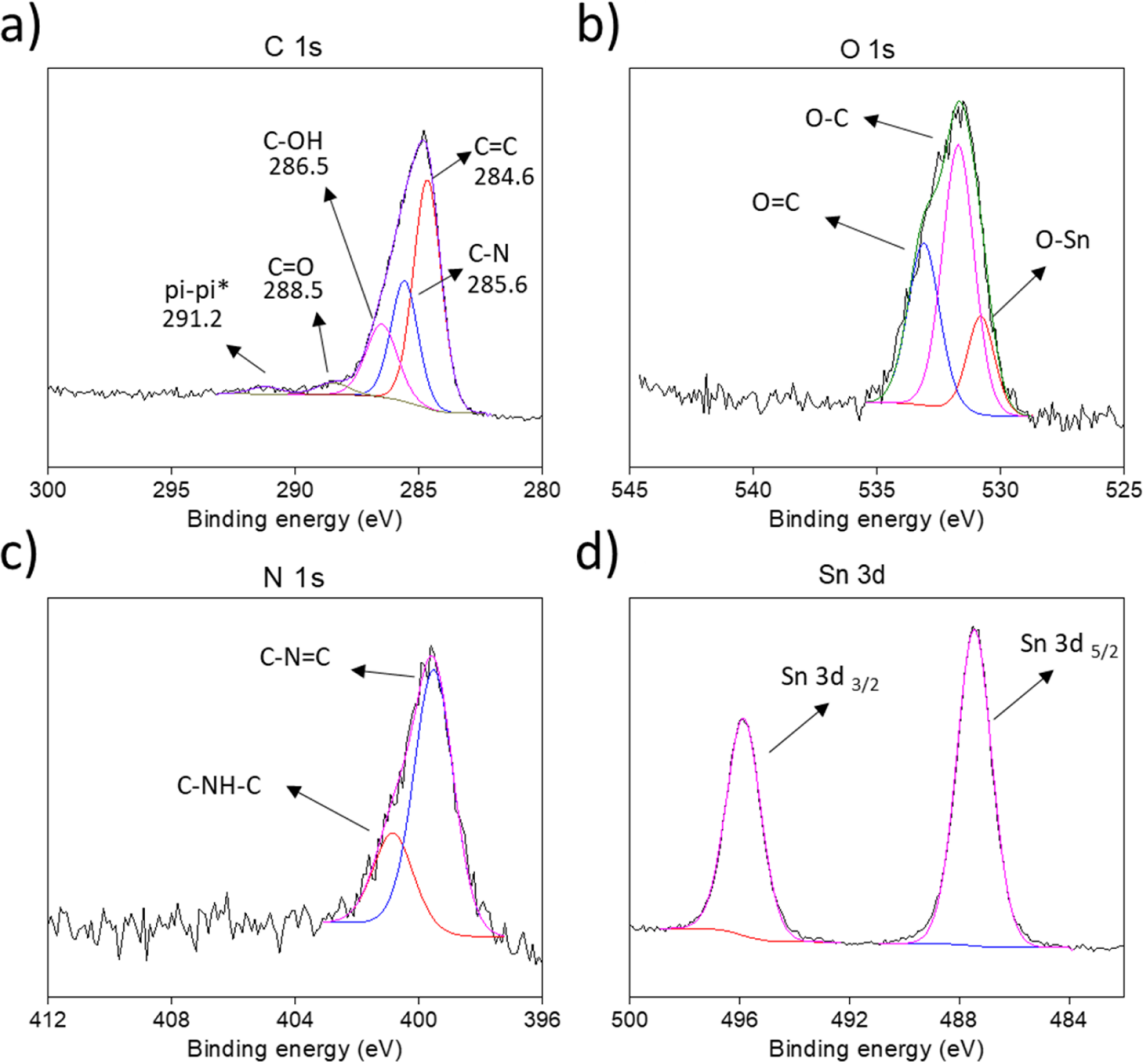
SnO_2_/PANI HR XPS spectra. Details of (a) C 1s, (b) O 1s, (c) N 1s, and (d) Sn 3d regions.

The low concentration of tin ions is also confirmed by the HR X-ray photoelectron spectroscopy (XPS) studies, which provide further information on the Sn atom chemical state. Figure 4 shows the HR XPS spectra of the SnO_2_/PANI sample. In Figure 4a, the C 1s signal is fitted with several contributions: the main ones of C=C (284.6 eV) and C–N (285.6 eV), coming from the polyaniline monomer^24^ and showing a correct ratio of, respectively, 4:2, C–OH (286.5 eV) and C=O (288.5 eV), respectively, from the quinone and hydroquinone moiety coming from hydrolysis of the immine group,^19,25^ and pi–pi* (291.2 eV) coming from the benzenoid moiety.^24^ In Figure 4b, the O 1s signal is fitted with the two contributions bonded to carbon, the O–C (531.7 eV) and O=C (533.1 eV), and a further signal attributable to O–Sn (530.8 eV) coming from SnO_2_ was also detected with XRD analysis.^26^ In Figure 4c, discriminating the chemical group in which nitrogen atoms are involved, it is possible to identify the different types of polyanilines, that is, the leucoemeraldine having only the benzenoid moiety (C–NH–C, 399.3 eV), emeraldine base having both benzenoid and quinonic moieties (C–N=C, 398.1 eV), and pernigraniline having further oxidized contributions.^25^ SnO_2_/PANI results to be an emeraldine base as expected, and also the theoretical stoichiometry value of 6:1, common among all the different PANIs, between C and N atoms, excluding the carbon bonded to oxygen, is coherently preserved.^25^Figure 4d shows the contributions of Sn 3d_3/2_ and _5/2_ (487.5 and 495.9 eV, respectively). Interestingly, the values obtained for such peaks are slightly higher compared to the ones registered in classic SnO_2_ samples^26−28^ suggesting a different environment for tin cations. Indeed, due to the extremely low size of clusters and the residual presence of chloride anions (Figure 2), the SnO_2_ nanoparticles appear to be Cl-doped in order to compensate the dense defects present on the SnO_2_ crystals,^21,22^ justifying also the higher binding energies registered in presence of such electronegativity atoms.

### Electrochemical Characterization

Due to the chemical nature as an ionomer of the polyaniline,^9^ the catalyst was drop casted onto a gas diffusion electrode without the presence of a binder for the fabrication of the cathode electrode. The catalyst ink was composed of catalyst powder mixed with Vulcan carbon nanoparticles with a weight ratio of 9:1 for a total powder density of 1 mg cm^–2^ on each electrode; the introduction of carbon nanoparticles in the slurry was intended to increase the overall electrical conductivity across the electrode.

**Figure 5:**
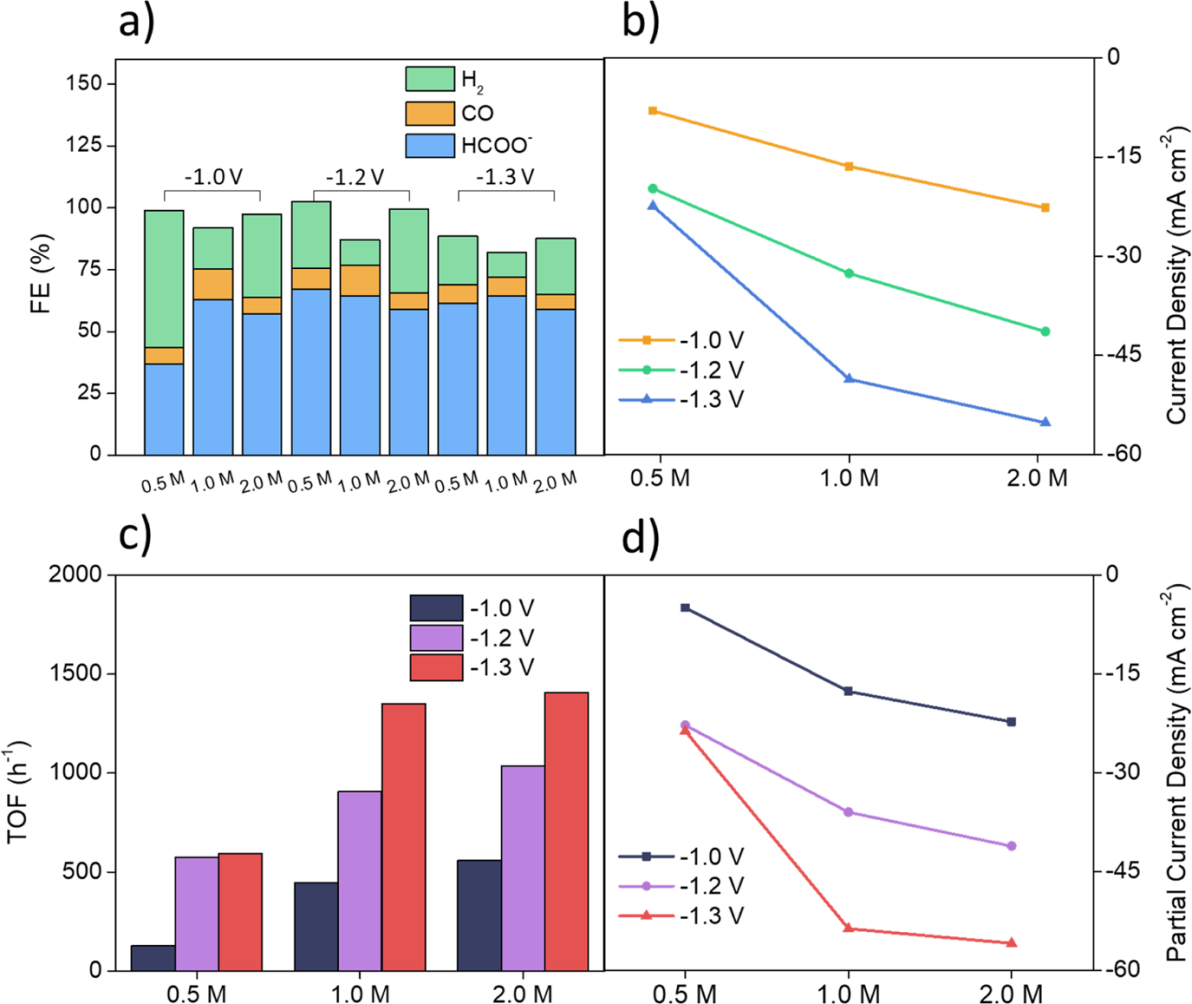
Chronoamperometry results in different combinations of electrolytes (0.5, 1.0, and 2.0 M of KHCO_3_) and applied potentials (−1.0, −1.2, and −1.3 V) vs RHE. (a) FEs, (b) current densities, (c) turnover frequencies, and (d) partial current densities for formate.

Different combinations of potentials and electrolytes were studied to optimize the HCOO^–^ selectivity as H_2_ and CO were produced in smaller quantities. Figure 5a shows the Faradaic efficiencies (FEs) for three different compositions of the electrolyte (0.5, 1.0, and 2.0 M of KHCO_3_) and applied potentials (−1.0, −1.2, and −1.3 V) with respect to the RHE, in parallel with the relative current densities recorded under the same conditions, as shown in Figure 5b. These values are obtained considering the average FEs of several minutes under stationary conditions for gaseous products and, for liquid products, considering the overall average FE_HCOO^–^_ obtained at the end 2 h experiment. All the further potentials will always refer to the RHE unless otherwise indicated. The relationship between potential and CO_2_RR suggests an operational applied potential close to −1.0 V.^10^ Indeed the differences among the potential chosen from −1.0 to −1.3 V reported in Figure 5a,b are rather small. In the current work, the highest average value of FE_HCOO^–^_, that is, 67.2%, is obtained in 0.5 M KHCO_3_ at −1.2 V due to the small amount of tin atoms present, that is, 7.0 wt % quantified by inductively coupled plasma mass spectrometry. The electrocatalysis of PANI alone showed only traces of CO_2_RR,^9^ so tin cations drastically change the selectivity of the overall catalysis. In Figure 5a, for each electrolyte, small differences in FE from −1.0 V to more negative potentials such as −1.2 and −1.3 V can be detected, with the exception of 0.5 M KHCO_3_. In the latter case, there is a significant increase in the selectivity of +30.4% switching from −1.0 to −1.2 V. Our interpretation is that the highest selectivity for CO_2_RR is theoretically achieved at −1.0 V versus RHE, as evidenced in 1.0 and 2.0 M KHCO_3_; however, the competition with the hydrogen evolution reaction (HER) in 0.5 M is too dominant, lowering the selectivity for CO_2_RR. Such problem is also still present for 1.0 and 2.0 M of KHCO_3_ but is drastically reduced by the pH buffer and the increased local production of OH^–^ in the Helmholtz layer. By shifting the potential from −1.0 to −1.2 V, the increase in current density leads to a higher local production of hydroxide anions^29^ as reported in eqs 1 and 2
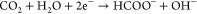
1
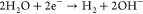
2

The increase in the local pH near the electrode is the main factor for the improved selectivity from −1.0 to −1.2 V in 0.5 M KHCO_3_. At equal pH, a higher FE for formate is reached, thanks to H^+^ depletion caused by OH^–^ ions produced near the electrode. In this electrolyte, the selectivity rises from 36.7 to 67.1% (Figure 5a) with a significant current density variation from 8.0 to 19.8 mA cm^–2^ (Figure 5b). By pushing the applied potential to −1.3 V in the 0.5 M KHCO_3_, no significant change in current density occurs, resulting in similar selectivity.

For the more concentrated electrolytes (1.0 and 2.0 M KHCO_3_), there is no comparable increase in selectivity from −1.0 to −1.2 V. This is because a more basic buffer is already present at these pH values and, at the same time, higher current densities already occur at −1.0 V due to a higher concentration of the inert salt. These two contributions lower the HER favoring the CO_2_RR. In fact, as shown in Figure 5b, the current density at −1.2 V in 0.5 M KHCO_3_ is 19.8 mA cm^–2^ (best formate selectivity condition), comparable to the values of 16.4 and 22.7 mA cm^–2^ already obtained at −1.0 V in 1.0 and 2.0 M KHCO_3_, respectively. These data suggest the complexity of the selectivity in the CO_2_RR since the current density also indirectly influences the CO_2_RR catalysis.^30^ The current density is mainly determined by the electrochemical setup, so different cell configurations would inevitably show different results even with the same catalyst. Considering the relative turnover frequencies (TOFs), calculated taking into account the total number of tin atoms in the catalyst, and the partial current densities for formate (respectively, Figure 5c,d), we can assume that the best operating conditions for the CO_2_RR electrolysis are certainly in the 1.0 M KHCO_3_ electrolyte, in which the TOF number is almost comparable with the one obtained in the more concentrated 2.0 M.

The SnO_2_/PANI catalyst shows interesting selectivities and current densities, which can be adjusted by the choice of electrolyte concentration. With the use of flow cell in the tests, a drastic increase in current density is observed. From the literature reported in the comparative Table 1, the highest FEs for the HCOO^–^ production (>90%) are observed in batch cell configuration or in setups capable of achieving limited current densities not exceeding 10 mA cm^–2^. Moreover, a well-established tendency in the literature to report the FEs registered in only few minutes of electrolysis overestimates the actual performances of the catalysts. Indeed, considering the small subset of works reporting a stability test of several hours, there is a huge mismatch between the FEs reported in the main scheme and the stability ones.

**Table 1:** Comparative Table Reporting Sn-Based Catalysts Resulting from the Previously Published Literature Together with the SnO_2_/PANI Highest-Selectivity and Highest-Current-Density Result Configuration

electrocatalyst	year	electrolyte	potential (V vs RHE)	*J*_HCOO^–^_	FE (%)	refs
Sn	1994	0.1 M NaHCO_3_	–1.48	5.0	88.4%	(31)
Sn/SnO_*x*_ thin film	2012	0.5 M NaHCO_3_	–0.70	0.7	40.0%	(32)
nano-SnO_2_/carbon black	2014	0.1 M KHCO_3_	–1.16	6.2	86.2%	(33)
nano-SnO_2_/graphene	2014	0.1 M KHCO_3_	–1.16	9.5	93.6%	(33)
Sn dendrite	2015	0.1 M NaHCO_3_	–1.36	17.1	71.6%	(34)
Sn quantum sheets/GO	2016	0.1 M NaHCO_3_	–1.16	21.1	89.0%	(35)
SnO_2_ porous NWs	2017	0.1 M NaHCO_3_	–0.80	4.8	80.0%	(36)
wire-in-tube SnO_2_	2018	0.1 M KHCO_3_	–0.99	3.8	63.0%	(37)
ultrasmall SnO NPs/C (2.6 nm)	2018	0.5 M KHCO_3_	–0.86	20.1	67.7%	(38)
ultrasmall SnO_2_ NPs (<5 nm)	2018	0.1 M KHCO_3_	–1.21	92.8	64.0%	(39)
chain-like mesoporous SnO_2_	2019	0.1 M KHCO_3_	–0.97	10.2	95.0%	(27)
mesoporous SnO_2_ nanosheets	2020	0.5 M NaHCO_3_	–0.90	14.0	83.0%	(40)
*m*-SnO_2_ NTs-350	2020	0.5 M KHCO_3_	–1.30	10.0	90.0%	(41)
<1 nm scale spaces on SnO_2_	2021	0.1 M KHCO_3_	–1.20	7.6	81.0%	(42)
Fe–SnO_2_	2022	0.1 M KHCO_3_	–0.89	6.0	41.0%	(43)
V_0_–SnO_2_	2022	0.5 M NaHCO_3_	–0.51	<5	92.4%	(44)
py-SnO_2_	2022	0.1 M KHCO_3_	–1.30	27.5	85.0%	(45)
Cl-doped SnO_2_/PANI	2022	1 M KHCO_3_	–1.20	23.5	72.0%	this work
Cl-doped SnO_2_/PANI	2022	2 M KHCO_3_	–1.3	32.6	59.1%	this work

**Figure 6:**
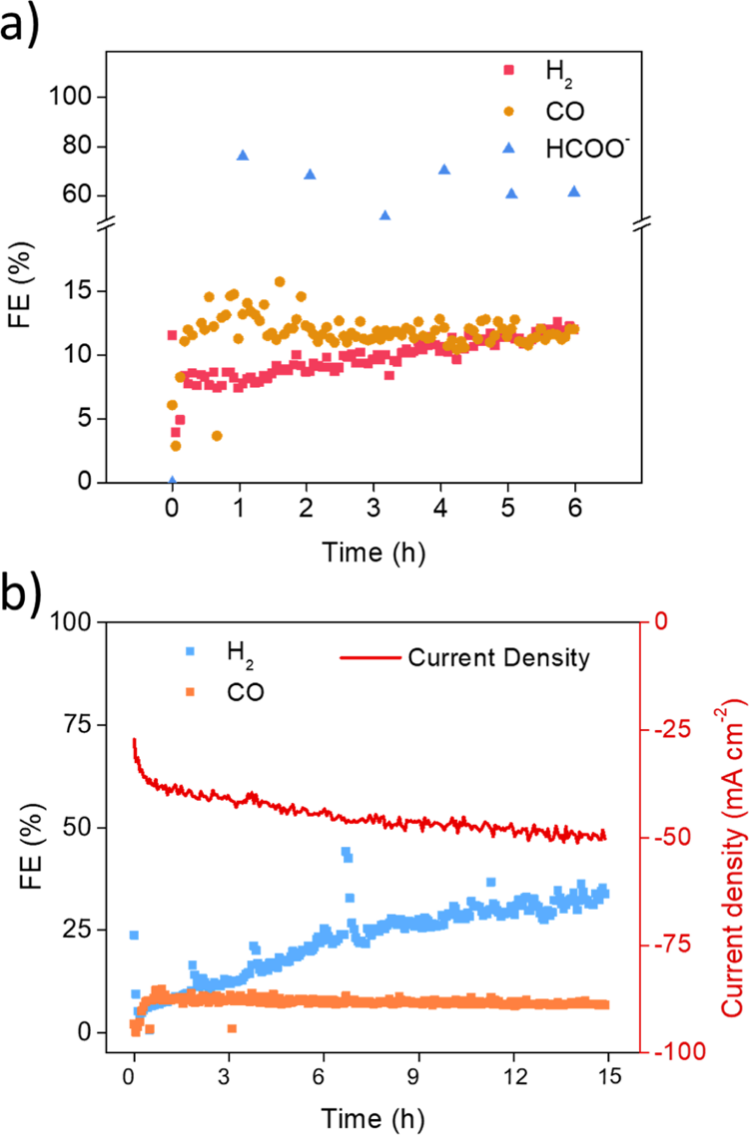
Chronoamperometry tests for stability evaluation. (a) HCOO^–^ production during time at −1.2 V vs RHE in 1.0 M KHCO_3_ and (b) current density, FE_H_2__ and FE_CO_ along 15 h at −1.2 V in 2.0 M KHCO_3_.

The SnO_2_/PANI catalyst was studied in the several electrolyte/potential combinations reporting an average value obtained for around 2 h of test, making the reported FE profoundly more significant. Considering the interesting current density registered, the actual FE for formate production is of compelling interest, especially considering the limited amount of catalyst employed. In addition, further efforts were made to study its behavior under continuous electrolysis conditions for several hours. A detailed stability test gives further information about how the selectivity changes as a function of the amount of formate produced, as shown in Figure 6a. A test performed at −1.2 V in 1.0 M KHCO_3_ shows a decreasing trend all along the 6 h of the test for the HCOO^–^ selectivity. After 1 hour of testing, the total FE_HCOO^–^_ reaches the highest value of 75.9% ever reached for the catalyst. The FE inevitably decreases from hour to hour until the last point on the 6th hour of 64.3%. Due to the nature of the setup, the electrolyte is mechanically recirculating through a peristaltic pump during the test. All along the experiment, HCOO^–^ concentration is increasing, as does the hydrogen evolution and the overall current density. To confirm this trend, an all-nightlong experiment was performed at −1.2 V in 2.0 M KHCO_3_. As shown in Figure 6b, the current density increases again parallel to the hydrogen evolution. Compared to the 6 h test in 1.0 M KHCO_3_ with the previously published work in 0.1 M KHCO_3_^10^ and the latter test of 16 h, it appears that the increase in hydrogen evolution and lowering of formate production are promoted by working in more concentrated electrolytes. The increase in conductivity and the intensification of the H_2_ evolution could be interpreted as a partial reduction to metallic tin sites, which are less selective toward the CO_2_RR. This information confirms 1.0 M KHCO_3_ as the best electrolyte for operating conditions.

## Conclusions

In conclusion, a binary catalyst consisting of a metal-doped polymer incorporating extremely low-size SnO_2_ nanoparticles was synthetized and tested for the electrochemical conversion of CO_2_ into formate. The amount of utilized metal is the strength of such an approach as the catalyst minimizes to the minimum necessary for the catalysis and relies on the organic part to build up and sustain the entire structure. The advantages of this approach also include the choice of cations, which can be switched according to the required application. The chemical nature of the polymer avoids the use of extremely expensive fluorinated binders to produce the electrodes, drastically lowering the price of the electrode preparation. The deposition of the low-dimensional catalyst on the polymer support, at the same time as its doping, represents a new and easy fabrication of scalable electrodes for the optimization of the catalyst used in the field of CO_2_RR.

## Experimental Methods

### Materials and Synthesis

All the chemicals were purchased from Merck without further purification, tin(IV) chloride pentahydrate (SnCl_4_·5H_2_O, 350.60 gmol^–1^ ≥98%), APS (228.20 gmol^–1^ ≥98%), Nafion 117 containing solution (5% in a mixture of lower aliphatic alcohols and water), NMP (99.5%), methanol (MeOH, 99.9%), sodium hydroxide (NaOH, 39.99 g mol^–1^ 98%), fuming hydrochloric acid (HCl, 36.5–38%), and bidistilled water (Milli-Q).

### ATR Fourier Transform Infrared Spectroscopy

ATR analysis was performed on a Bruker Tensor II in transmission mode placing the pure sample on the surface of the crystal. The analysis was performed in the range of 4000–400 cm^–1^.

### X-ray Diffraction

Patterns were recorded in Bragg–Brentano symmetric geometry by using a PANalyti-cal X’Pert Pro instrument (Cu Kα radiation, 40 kV and 30 mA) equipped with an X’Celerator detector.

### X-Ray Photoelectron Spectroscopy

The analysis was performed with a PHI 5000 Versaprobe spectrometer (Physical Electronics), equipped with a monochromatic Al K-alpha X-ray source (1486.6 eV). Surface charge compensation was obtained with a combined system, based on an electron gun and Ar^+^ ion gun. Survey and HR spectra were acquired using pass energy values of 187.85 and 23.50 eV, respectively. The calibration of the binding energy scale was obtained by setting the C–C component of the C 1s region to 284.6 eV. Casa XPS software was used for the analysis of the experimental data. The Shirley background function was subtracted from HR spectra to remove the background signal.^46^

### Field-Emission Scanning Electron Microscopy

The images were recorded on a ZEISS Supra 40 field-emission scanning electron microscope with the following configuration: SE2 detector for secondary-electron imaging, BSE detector for back-scattered electron imaging, Si(Li) Oxford Instruments detector for EDX Spectroscopy.

### Electrochemical Setup

The as-prepared catalysts were mixed together to Vulcan carbon nanoparticles with a weight ratio of 9:1 and were dispersed in 160 μL of iPrOH by sonication. The slurry was then coated onto a carbon paper (GDL; SIGRACET 28BC, SGL Technologies) in order to enable the electrochemical evaluation of the powder-like materials toward the CO_2_RR. The powder deposition reaches the 1 mg cm^–2^ loading on each electrode.

### Cyclic Voltammetry

The CVs of the SnO_2_/PANI were performed in a three-electrode monocell at RT with a Metrohm Multi Autolab/M101 potentiostat. The working electrode was a catalyst-coated carbon paper with a geometric area of 0.15 cm^2^ where the catalyst loading was of 1 mg cm^–2^. A Pt wire was used as the counter electrode and Ag/AgCl (3 M NaCl) was used as the reference electrode. The CVs were performed with a scan rate of 10 mV s^–1^ for several cycles and reporting only the last run. The measurements were performed separately in N_2_- and CO_2_-saturated/purged (5 mL min^–1^) 0.1 M KHCO_3_ aqueous solution. All the potentials refer to RHE in this work, in which the conversion followed the equation: *E* (vs RHE) = *E* (vs Ag/AgCl) + 0.197 V + 0.0591 × pH.

### Chronoamperometry

Tests were performed with a CHI 760D (CH Instruments, Inc.) potentiostat in a customized two-compartment cell (ElectroCell) with a proton-exchange membrane (Nafion Membrane N117, Ion Power). Both cathodic and anodic compartments were connected to a mechanical pump which promotes the circulation of the electrolyte at 2 mL min^–1^. A catalyst-coated carbon paper (GDL28 SIGRACET 28BC, SGL Technologies) of 1.5 cm^2^ was used as the working electrode, a 4 cm^2^ Pt foil as the counter, and Ag/AgCl (1 mm, leak-free LF-1) as the reference. Gas-phase products were analyzed on-line by micro gas chromatography (μGC, Fusion, INFICON) with two channels containing a 10 m Rt-Msieve 5A column and an 8 m Rt-Q-Bond column, respectively. Both channels were equipped with a micro thermal conductivity detector (micro-TCD). The inlet of the μGC equipment was connected to the cathodic side of the electrochemical cell through a GENIE filter to remove the humidity from the gas. During the CA measurements, a constant CO_2_ flow rate of 25 mL min^–1^ was maintained on the back site of the electrode (gas-diffusion layer) to assure the reactant supply and to bring the gaseous products to the μGC. Liquid products were analyzed by high-performance liquid chromatography (Thermo Scientific Ultimate3000 HPLC) with a UV–vis Detector set at 210 nm by using a ReproGel (300 × 8 mm) column with 9.0 mM H_2_SO_4_ (flow rate of 1.0 mL min^–1^) as the mobile phase. The FE for each product was calculated by dividing the coulombs needed to produce the actual determined amount of this product by the total coulombs consumed during the corresponding reduction period of each measurement.

## References

[ref1] Bushuyev O. S.; De Luna P.; Dinh C. T.; Tao L.; Saur G.; van de Lagemaat J.; Kelley S. O.; Sargent E. H. What Should We Make with CO2 and How Can We Make It?. Joule 2018, 2, 825–832. 10.1016/j.joule.2017.09.003.

[ref2] De Luna P.; Hahn C.; Higgins D.; Jaffer S. A.; Jaramillo T. F.; Sargent E. H. What Would It Take for Renewably Powered Electrosynthesis to Displace Petrochemical Processes?. Science 2019, 364, 6438 10.1126/science.aav3506. 31023896

[ref3] Jouny M.; Luc W.; Jiao F. General Techno-Economic Analysis of CO2 Electrolysis Systems. Ind. Eng. Chem. Res. 2018, 57, 2165–2177. 10.1021/acs.iecr.7b03514.

[ref4] Weekes D. M.; Salvatore D. A.; Reyes A.; Huang A.; Berlinguette C. P. Electrolytic CO2 Reduction in a Flow Cell. Acc. Chem. Res. 2018, 51, 910–918. 10.1021/acs.accounts.8b00010. 29569896

[ref5] Sheet S. D. Formic Acid Formic Acid. ICIS Chem. Bus. 2017, 1, 1–12.

[ref6] Ma Z.; Legrand U.; Pahija E.; Tavares J. R.; Boffito D. C. From CO2to Formic Acid Fuel Cells. Ind. Eng. Chem. Res. 2021, 60, 803–815. 10.1021/acs.iecr.0c04711.

[ref7] Navarro-Jaén S.; Virginie M.; Bonin J.; Robert M.; Wojcieszak R.; Khodakov A. Y. Highlights and Challenges in the Selective Reduction of Carbon Dioxide to Methanol. Nat. Rev. Chem. 2021, 5, 564 10.1038/s41570-021-00289-y. 37117584

[ref8] Liu A.; Gao M.; Ren X.; Meng F.; Yang Y.; Gao L.; Yang Q.; Ma T. Current Progress in Electrocatalytic Carbon Dioxide Reduction to Fuels on Heterogeneous Catalysts. J. Mater. Chem. A 2020, 8, 3541–3562. 10.1039/c9ta11966c.

[ref9] Sassone D.; Zeng J.; Fontana M.; Sacco A.; Farkhondehfal M. A.; Periolatto M.; Pirri C. F.; Bocchini S. Polymer-Metal Complexes as Emerging Catalysts for Electrochemical Reduction of Carbon Dioxide. J. Appl. Electrochem. 2021, 51, 1301–1311. 10.1007/s10800-021-01585-7.

[ref10] Nguyen T. N.; Salehi M.; Le Q.; Seifitokaldani A.; Dinh C. T. Fundamentals of Electrochemical CO2Reduction on Single-Metal-Atom Catalysts. ACS Catal. 2020, 10, 10068–10095. 10.1021/acscatal.0c02643.

[ref11] Yang H.; Wu Y.; Li G.; Lin Q.; Hu Q.; Zhang Q.; Liu J.; He C. Scalable Production of Efficient Single-Atom Copper Decorated Carbon Membranes for CO2 Electroreduction to Methanol. J. Am. Chem. Soc. 2019, 141, 12717–12723. 10.1021/jacs.9b04907. 31348650

[ref12] Rotundo L.; Garino C.; Priola E.; Sassone D.; Rao H.; Ma B.; Robert M.; Fiedler J.; Gobetto R.; Nervi C. Electrochemical and Photochemical Reduction of CO 2 Catalyzed by Re(I) Complexes Carrying Local Proton Sources. Organometallics 2019, 38, 1351 10.1021/acs.organomet.8b00588.

[ref13] Ren S.; Joulié D.; Salvatore D.; Torbensen K.; Wang M.; Robert M.; Berlinguette C. P. Molecular Electrocatalysts Can Mediate Fast, Selective CO2 Reduction in a Flow Cell. Science 2019, 365, 367–369. 10.1126/science.aax4608. 31346062

[ref14] Wang M.; Torbensen K.; Salvatore D.; Ren S.; Joulié D.; Dumoulin F.; Mendoza D.; Lassalle-Kaiser B.; Işci U.; Berlinguette C. P.; Robert M. CO2 Electrochemical Catalytic Reduction with a Highly Active Cobalt Phthalocyanine. Nat. Commun. 2019, 10, 3602 10.1038/s41467-019-11542-w. 31399585 PMC6689005

[ref15] Zeng J.; Jagdale P.; Lourenço M. A. O.; Farkhondehfal M. A.; Sassone D.; Bartoli M.; Pirri C. F. Biochar-Supported BiOx for Effective Electrosynthesis of Formic Acid from Carbon Dioxide Reduction. Crystals 2021, 11, 363 10.3390/cryst11040363.

[ref16] Su P.; Xu W.; Qiu Y.; Zhang T.; Li X.; Zhang H. Ultrathin Bismuth Nanosheets as a Highly Efficient CO2Reduction Electrocatalyst. ChemSusChem 2018, 11, 848–853. 10.1002/cssc.201702229. 29323463

[ref17] Zhang G.; Zhao Z. J.; Cheng D.; Li H.; Yu J.; Wang Q.; Gao H.; Guo J.; Wang H.; Ozin G. A.; Wang T.; Gong J. Efficient CO2 Electroreduction on Facet-Selective Copper Films with High Conversion Rate. Nat. Commun. 2021, 12, 1–11. 10.1038/s41467-021-26053-w. 34593804 PMC8484611

[ref18] Liu L.; Corma A. Evolution of Isolated Atoms and Clusters in Catalysis. Trends Chem. 2020, 2, 383–400. 10.1016/j.trechm.2020.02.003.

[ref19] Golczak S.; Kanciurzewska A.; Fahlman M.; Langer K.; Langer J. Comparative XPS surface study of polyaniline thin films. Solid State Ionics 2008, 179, 2234–2239. 10.1016/j.ssi.2008.08.004.

[ref20] Chiolerio A.; Bocchini S.; Crepaldi M.; Bejtka K.; Pirri C. F. Bridging Electrochemical and Electron Devices: Fast Resistive Switching Based on Polyaniline from One Pot Synthesis Using FeCl3 as Oxidant and Co-Doping Agent. Synth. Met. 2017, 229, 72–81. 10.1016/j.synthmet.2017.05.001.

[ref21] Gong W.; Guo H.; Zhang H.; Yang J.; Chen H.; Wang L.; Hao F.; Niu X. Chlorine-Doped SnO2hydrophobic Surfaces for Large Grain Perovskite Solar Cells. J. Mater. Chem. C 2020, 8, 11638–11646. 10.1039/d0tc00515k.

[ref22] Ren X.; Liu Y.; Lee D. G.; Kim W. B.; Han G. S.; Jung H. S.; Liu S. Chlorine-modified SnO 2 Electron Transport Layer for High-efficiency Perovskite Solar Cells. InfoMat 2020, 2, 401–408. 10.1002/inf2.12059.

[ref23] Liang J.; Chen Z.; Yang G.; Wang H.; Ye F.; Tao C.; Fang G. Achieving High Open-Circuit Voltage on Planar Perovskite Solar Cells via Chlorine-Doped Tin Oxide Electron Transport Layers. ACS Appl. Mater. Interfaces 2019, 11, 23152–23159. 10.1021/acsami.9b03873. 31184462

[ref24] Tantawy H. R.; Kengne B. A. F.; McIlroy D. N.; Nguyen T.; Heo D.; Qiang Y.; Aston D. E. X-Ray Photoelectron Spectroscopy Analysis for the Chemical Impact of Solvent Addition Rate on Electromagnetic Shielding Effectiveness of HCl-Doped Polyaniline Nanopowders. J. Appl. Phys. 2015, 118, 175501 10.1063/1.4934851.

[ref25] Mohtasebi A.; Chowdhury T.; Hsu L. H. H.; Biesinger M. C.; Kruse P. Interfacial Charge Transfer between Phenyl-Capped Aniline Tetramer Films and Iron Oxide Surfaces. J. Phys. Chem. C 2016, 120, 29248–29263. 10.1021/acs.jpcc.6b09950.

[ref26] Kwoka M.; Ottaviano L.; Passacantando M.; Santucci S.; Czempik G.; Szuber J. XPS Study of the Surface Chemistry of L-CVD SnO2 Thin Films after Oxidation. Thin Solid Films 2005, 490, 36–42. 10.1016/j.tsf.2005.04.014.

[ref27] Bejtka K.; Zeng J.; Sacco A.; Castellino M.; Hernández S.; Farkhondehfal M. A.; Savino U.; Ansaloni S.; Pirri C. F.; Chiodoni A. Chainlike Mesoporous SnO2 as a Well-Performing Catalyst for Electrochemical CO2 Reduction. ACS Appl. Energy Mater. 2019, 2, 3081–3091. 10.1021/acsaem.8b02048.

[ref28] Stranick M. A.; Moskwa A. SnO2 by XPS. Surf. Sci. Spectra 1993, 2, 50–54. 10.1116/1.1247724.

[ref29] Sun D.; Chen Y. Electrode Kinetics of CO2 Electroreduction; CRC Press, 2016.

[ref30] Huang J. E.; Li F.; Ozden A.; Sedighian Rasouli A. S.; García de Arquer F. P. G.; Liu S.; Zhang S.; Luo M.; Wang X.; Lum Y.; Xu Y.; Bertens K.; Miao R. K.; Dinh C. T.; Sinton D.; Sargent E. H. CO2 Electrolysis to Multicarbon Products in Strong Acid. Science 2021, 372, 1074–1078. 10.1126/science.abg6582. 34083485

[ref31] Hori Y.; Wakebe H. H. I.; Tsukamoto T.; Koga O. Electrocatalytic Process of CO Selectivity in Electrochemical Reduction of CO2 at Metal Electrodes in Aqueous Media. Electrochim. Acta 1994, 39, 1833–1839. 10.1016/0013-4686(94)85172-7.

[ref32] Chen Y.; Kanan M. W. Tin Oxide Dependence of the CO 2 Reduction Efficiency on Tin Electrodes and Enhanced Activity for Tin/Tin Oxide Thin-Film Catalysts. J. Am. Chem. Soc. 2012, 134, 1986–1989. 10.1021/ja2108799. 22239243

[ref33] Zhang S.; Kang P.; Meyer T. J. Nanostructured Tin Catalysts for Selective Electrochemical Reduction of Carbon Dioxide to Formate. J. Am. Chem. Soc. 2014, 136, 1734–1737. 10.1021/ja4113885. 24417470

[ref34] Won D. H.; Choi C. H.; Chung J.; Chung M. W.; Kim E. H.; Woo S. I. Rational Design of a Hierarchical Tin Dendrite Electrode for Efficient Electrochemical Reduction of CO2. ChemSusChem 2015, 8, 3092–3098. 10.1002/cssc.201500694. 26219092

[ref35] Lei F.; Liu W.; Sun Y.; Xu J.; Liu K.; Liang L.; Yao T.; Pan B.; Wei S.; Xie Y. Metallic Tin Quantum Sheets Confined in Graphene toward High-Efficiency Carbon Dioxide Electroreduction. Nat. Commun. 2016, 7, 12697 10.1038/ncomms12697. 27585984 PMC5025773

[ref36] Kumar B.; Atla V.; Brian J. P.; Kumari S.; Nguyen T. Q.; Sunkara M.; Spurgeon J. M. Reduced SnO2 Porous Nanowires with a High Density of Grain Boundaries as Catalysts for Efficient Electrochemical CO2-into-HCOOH Conversion. Angew. Chem., Int. Ed. 2017, 56, 3645–3649. 10.1002/anie.201612194. 28229519

[ref37] Fan L.; Xia Z.; Xu M.; Lu Y.; Li Z. 1D SnO2 with Wire-in-Tube Architectures for Highly Selective Electrochemical Reduction of CO2 to C1 Products. Adv. Funct. Mater. 2018, 28, 1706289 10.1002/adfm.201706289.

[ref38] Gu J.; Héroguel F.; Luterbacher J.; Hu X. Densely Packed, Ultra Small SnO Nanoparticles for Enhanced Activity and Selectivity in Electrochemical CO2 Reduction. Angew. Chem., Int. Ed. 2018, 57, 2943–2947. 10.1002/anie.201713003. 29356272

[ref39] Liang C.; Kim B.; Yang S.; Yang Liu Y.; Francisco Woellner C.; Li Z.; Vajtai R.; Yang W.; Wu J.; Kenis P. J. A.; Ajayan P. M. High Efficiency Electrochemical Reduction of CO2 beyond the Two-Electron Transfer Pathway on Grain Boundary Rich Ultra-Small SnO2 Nanoparticles. J. Mater. Chem. A 2018, 6, 10313–10319. 10.1039/c8ta01367e.

[ref40] Han N.; Wang Y.; Deng J.; Zhou J.; Wu Y.; Yang H.; Ding P.; Li Y. Self-Templated Synthesis of Hierarchical Mesoporous SnO 2 Nanosheets for Selective CO 2 Reduction. J. Mater. Chem. A 2019, 7, 1267–1272. 10.1039/c8ta10959a.

[ref41] Wei F.; Wang T.; Jiang X.; Ai Y.; Cui A.; Cui J.; Fu J.; Cheng J.; Lei L.; Hou Y.; Liu S. Controllably Engineering Mesoporous Surface and Dimensionality of SnO2 toward High-Performance CO2 Electroreduction. Adv. Funct. Mater. 2020, 30, 2002092 10.1002/adfm.202002092.

[ref42] Kim M. K.; Lee H.; Won J. H.; Sim W.; Kang S. J.; Choi H.; Sharma M.; Oh H. S.; Ringe S.; Kwon Y.; Jeong H. M. Design of Less than 1 Nm Scale Spaces on SnO2 Nanoparticles for High-Performance Electrochemical CO2 Reduction. Adv. Funct. Mater. 2022, 32, 2270048 10.1002/adfm.202270048.

[ref43] Savino U.; Sacco A.; Bejtka K.; Castellino M.; Farkhondehfal M. A.; Chiodoni A.; Pirri F.; Tresso E. Well Performing Fe-SnO2 for CO2 Reduction to HCOOH. Catal. Commun. 2022, 163, 106412 10.1016/j.catcom.2022.106412.

[ref44] Liu G.; Li Z.; Shi J.; Sun K.; Ji Y.; Wang Z.; Qiu Y.; Liu Y.; Wang Z.; Hu P. A. Black Reduced Porous SnO2 Nanosheets for CO2 Electroreduction with High Formate Selectivity and Low Overpotential. Appl. Catal., B 2020, 260, 118134 10.1016/j.apcatb.2019.118134.

[ref45] Zhang Y.; Xu H.; Niu D.; Zhang X.; Zhang Y. Pyridine Grafted on SnO2-Loaded Carbon Nanotubes Acting as Cocatalyst for Highly Efficient Electroreduction of CO2. ChemSusChem 2021, 14, 2769–2779. 10.1002/cssc.202100541. 33855812

[ref46] Shirley D. A. High-Resolution x-Ray Photoemission Spectrum of the Valence Bands of Gold. Phys. Rev. B: Solid State 1972, 5, 4709–4714. 10.1103/physrevb.5.4709.

